# Protocol to study ductal progenitor-like cells from the adult human pancreas using 3D suspension and methylcellulose-based culture systems

**DOI:** 10.1016/j.xpro.2025.103847

**Published:** 2025-05-26

**Authors:** Heather N. Zook, Janine C. Quijano, Jose A. Ortiz, Cecile Donohue, Neslihan Erdem, Hsun Teresa Ku

**Affiliations:** 1 Department of Translational Research and Cellular Therapeutics, Arthur Riggs Diabetes and Metabolism Research Institute, City of Hope, Duarte, CA 91010, USA; 2 Irell & Manella Graduate School of Biological Sciences, Beckman Research Institute, City of Hope, Duarte, CA 91010, USA

**Keywords:** Cell Biology, Cell culture, Organoids

## Abstract

Primary human ductal progenitor-like cells derived from donated pancreas have the potential to serve as a source of therapeutic insulin-producing beta cells for the treatment of diabetes. Here, we present a protocol for studying ductal progenitor-like cells using a good manufacturing practice (GMP)-compatible 3D suspension culture system and a methylcellulose- and Matrigel-based 3D colony assay. We describe steps for dispersing and cryopreserving human pancreatic cells and initiating and maintaining cultures. We then detail how to prepare ductal spheroids or colonies for downstream applications.

For complete details on the use and execution of this protocol, please refer to Zook et al.^1^ and Quijano et al.^2^

## Highlights

• Stepwise protocol to dissociate and cryopreserve primary human exocrine tissues
• Instructions to maintain human ductal cells/spheroids using 3D suspension culture
• Steps to prepare and use 3D methylcellulose-based colony assay to generate colonies
• Steps for analysis and processing of spheroids and colonies

## Abstract (editor highlights)

Publisher’s note: Undertaking any experimental protocol requires adherence to local institutional guidelines for laboratory safety and ethics.

## Graphical abstract

**Figure:**
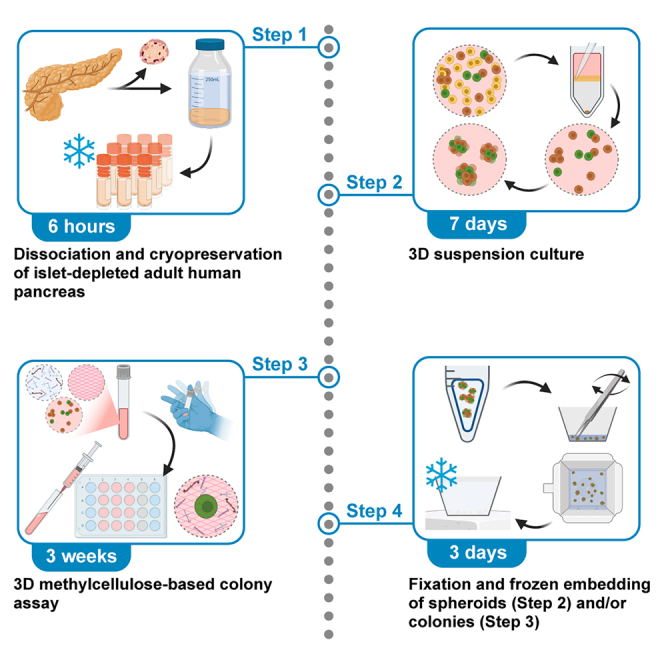

## Before you begin

The protocols below describe two 3-dimensional (D) culture platforms designed by our laboratory to study the survival, expansion, self-renewal, and differentiation of human ductal progenitor-like cells isolated from cadaveric pancreases. It should be noted that in the above-mentioned culture platforms, Matrigel is used at a concentration of 5% (v/v). This is different from other 3D organoid culture platforms,^3^^,^^4^ which use ∼100% (v/v) Matrigel to embed ductal cells before adding the liquid medium for culture. It should also be noted that, in the report by Zook et al. 2024,^1^ we have identified 6 growth factors in the 3D suspension culture platform necessary to maintain the survival of human ductal progenitor-like cells in the absence of Matrigel and fetal calf serum, but in the presence of Knockout Serum Replacement. These 6 factors are nicotinamide, epithelial growth factor, Noggin, gastrin, R-spondin 1, and A83-01. Thus, our 3D suspension culture platform serves as a foundation upon which additional signals necessary for the activation of ductal progenitor-like cells can be identified. For instance, we reported that extracellular matrix proteins (Matrigel and specifically collagen IV) are necessary to activate human ductal progenitor-like cells via integrin receptors,^1^ resulting in their improved survival and proliferation.

One critical consideration before embarking on obtaining human cadaveric pancreatic tissue for study is to acquire an approval from the Institutional Review Board (IRB). However, because the donor tissues are delivered to the research investigators as deidentified, which does not meet the definition of human subject research as set forth at (45 CFR 46.102(e)), the IRB determined the research “not human subjects research”.

### Institutional permissions

The research activities described below involved the use of adult cadaveric pancreases, which were obtained by the Southern California Islet Cell Resource (SC-ICR) Center at the City of Hope. SC-ICR isolated the endocrine islets from the exocrine tissues. The specific policies and standard operating procedures for islet isolation can be found in the information provided by the integrated islet distribution program (IIDP) (https://iidp.coh.org/). Subsequently, the exocrine tissues were delivered from SC-ICR to the laboratory of H.T.K. for research purposes. This process was reviewed and approved by the IRB at the City of Hope as Non-Human Subjects Research (Protocol# 23728). All methods were performed in accordance with the relevant guidelines and regulations.

### Preparation of cryopreserved adult human islet-depleted exocrine cells

**Timing: 4–6 h**

This step details how to prepare a single-cell suspension from a cadaveric human exocrine tissue that has had the islets previously removed. It should be noted that although human donors were screened against a number of infectious diseases by the SC-ICR, universal precautions and aseptic technique should be practiced while handling in the laboratory.

1. Preparation of tissue:
  a. Receive islet-depleted pancreatic exocrine tissue from the SC-ICR and keep on ice (4°C).
    ***Note:*** We receive tissue stored in either UW solution^5^ or PIM(T) media (Prodo Laboratory) (https://doi.org/10.17504/protocols.io.bhdpj25n). Tissue may be stored at 4°C protected from light until use for up to 5 days post-islet isolation.
  b. Ensure liquid nitrogen for controlled rate freezer is available, if needed (see materials and equipment section for more information).
2. Preparation of reagents (see materials and equipment):
  a. PBS: Dulbecco’s phosphate-buffered saline (PBS, 21-031-CV, Corning).
    ***Note:*** All “PBS” mentioned throughout this protocol refers to Dulbecco’s phosphate-buffered saline.
  b. PBS/BSA/PS: PBS, 0.1% (w/v) Bovine Serum Albumin (BSA, A8412, Sigma-Aldrich), and 1x Penicillin-Streptomycin (PS, 15140-122, Gibco).
  c. DNase1 solution: 1 MU/mL DNase1 (260913-10MU, EMD Millipore) in PBS/BSA/PS.
  d. PBS/BSA/PS/DNase1: PBS/BSA/PS and 2,000 U/mL DNase1.
  e. Collagenase B solution: 100 mg/mL Collagenase B (11088831001, Sigma-Aldrich) in PBS/BSA/PS.
    ***Note:*** The volumes to prepare for each reagent are dependent on the number of aliquots to be made in step 3c of this protocol. For each aliquot, please prepare: 30 mL PBS, 45 mL PBS/BSA/PS, 200 μL DNase1 solution, 45 mL PBS/BSA/PS/DNase1, and 600 μL Collagenase B solution.
  f. CryoStor CS10 (210102, Biolife Solutions) (recommended at least 30 mL per tissue).
3. Dissociation of islet-depleted pancreatic exocrine tissue (Figure 1A):
  **CRITICAL:** At all times during the following procedure, aseptic technique is essential. Make sure sterility is maintained when handling the tissue outside of the tissue culture hood by keeping the lid screwed on the 50 mL conical tubes and by sterilizing the tubes using ethanol when re-entering the tissue culture hood.
  a. Aspirate storage media until the tissue volume relative to the total volume is about 1:5 (Figure 1B).
    ***Note:*** The islet-depleted exocrine tissue should be well separated (e.g. it tends to form a yellowish structure) from the clear storage media. It is recommended to record the tissue volume. The IIDP typically adds enough storage media such that the tissue volume relative to the total volume is about 1:10.
  b. Resuspend the tissue on ice by gently inverting the bottle several times and pipetting slowly using a serological pipette (A.K.A. electronic pipette, or pipette aid) until homogenous.
  c. Aliquot 10 mL resuspended tissue into 50 mL tubes (Figure 1B).
    ***Note:*** The resulting number of tissue aliquots will vary depending on the volume of tissue initially received. Each 10 mL aliquot of resuspended tissue is comprised of ∼2 mL of the islet-depleted exocrine tissue plus ∼8 mL storage media (such as PIM(T) storage media). Thus, if 20 mL of tissue is received (step 1a), it is expected that 10 aliquots will be made in this step following resuspension in storage media.
  d. Pour cold PBS to bring volume up to 40 mL total and add 80 μL DNase1 stock solution (2,000 U/mL final concentration).
  e. Invert tubes gently 5–10 times to mix and let tubes stand on ice (4°C) for 10 min for gravity to pull down the tissue.
    i. If tissue does not settle after 10 min, keep on ice (4°C) for an additional 10 min.
    ii. If tissue has not settled enough to form a pellet, or if an opaque or cloudy supernatant is observed after 20 min total time, see problem 1.
  f. Meanwhile, prepare 45 mL PBS/BSA/PS/DNase1 solution per tube of tissue by diluting DNase1 stock solution at 1:500 (2,000 U/mL final concentration).
    ***Note:*** PBS/BSA/PS/DNase1 can be kept at 22°C for the remainder of the protocol.
  g. To minimize cell loss, aspirate supernatant without touching the loose cell pellet.
  h. Pour PBS/BSA/PS/DNase1 to bring volume to 25 mL total, then add 600 μL Collagenase B stock solution (2.4 mg/mL final concentration).
    ***Note:*** Recommend handling no more than six 50 mL tubes at one time by a single person.
  i. Mix by 5–6 gentle inversions, then place the tubes in 37°C water bath for 10 min. During 10 min incubation, gently invert tubes 5–6 times every 3–4 min.
  j. After 10 min, mechanically disperse cell clusters by drawing the cell solution through a 16½ G needle mounted on a 10 mL syringe (302995, BD Biosciences) and dispensing along the side of the tube 7 times.
    **CRITICAL:** During mechanical dispersal, angle the needle tip 45° relative to the side of the 50 mL tube, and do not apply too much pressure. Too much pressure could damage acinar cells, leading to leakage of digestive enzymes that could harm the exocrine cells. However, too little pressure will not allow for adequate dispersal. Avoid making bubbles as much as possible.
  k. Repeat steps 3i-3j once more.
  l. To stop Collagenase B digestion, pour PBS/BSA/PS/DNase1 up to 45 mL total volume and mix by 5-6 gentle inversions.
    ***Note:*** Target a maximum time in Collagenase B solution at 30 min.
  m. Centrifuge at 300–400 x g for 5 min.
    ***Note:*** All centrifugation steps for live cells conducted in this protocol should be done at ambient (∼22°C) temperature.

**Figure 1:**
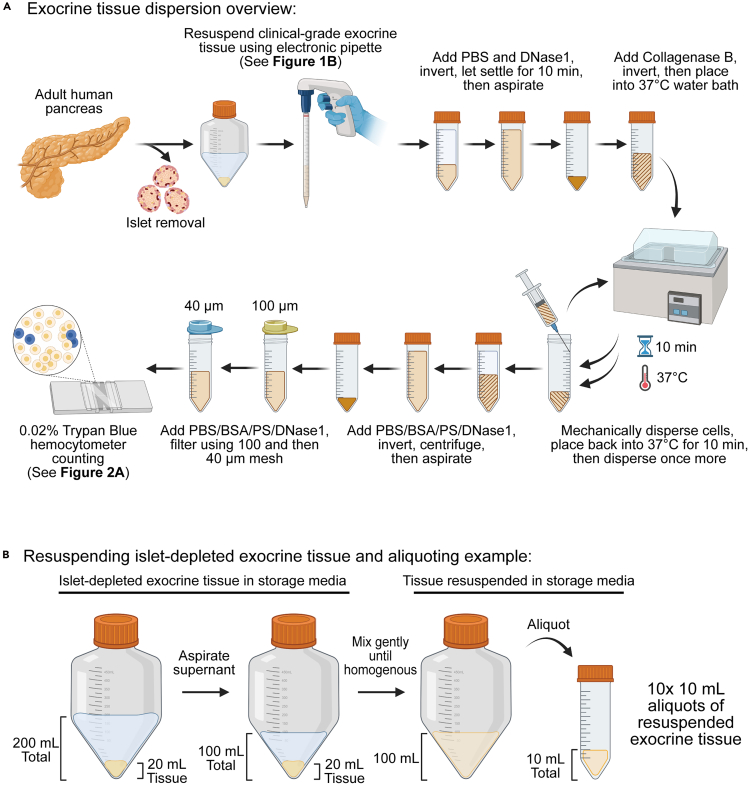
Dissociation of islet-depleted exocrine tissue derived from adult human pancreas
4. Filter the dispersed pancreatic cells (Figure 1A).
  a. Remove supernatant being careful of the soft pellets.
    ***Note:*** Make sure the supernatant is mostly clear. A little cloudy is acceptable if a clear pellet is visible. Lack of a clear pellet indicates that most of the cells have likely died (see problem 1).
    ***Note:*** It is acceptable to leave a thin layer of supernatant on top.
  b. Add 3 mL PBS/BSA/PS/DNase1 using a p1000 pipette tip and resuspend cells gently.
  c. Add 10 μL DNase1 stock solution, then another 2–3 mL PBS/BSA/PS/DNase1.
    ***Note:*** The final concentration of DNase1 in the cell solution will be about 4,000 U/mL. Using additional DNase1 at this step assists with reconstitution of the cell pellet for quicker filtration compared to not adding the extra DNase1.
  d. On ice (4°C), filter cells through a 100 μm mesh filter (22-363-549, Fisher Scientific) into 50 mL tubes.
    ***Note:*** If multiple 50 mL tubes (e.g. 6 tubes) with dissociated cell suspensions were handled, one can combine them during this step.
    **CRITICAL:** It is important to minimize the total amount of time the dissociated cells spend in the filtering steps to prevent re-aggregation. It is thus recommended that at least two people filter cells in parallel, especially when working with multiple tubes of dissociated cell suspensions.
  e. Once all the dissociated cells are filtered through 100 μm mesh, add 2 μL DNase1 stock solution per mL of cells and mix by 3–4 gentle inversions.
  f. On ice (4°C), filter cells through two to four 40 μm mesh filters (22-363-547, Fisher Scientific) to obtain a mostly single cell suspension.
    ***Note:*** If cells run too slowly or clog the filter, please see problem 2.
  g. Pool cells into one or two 50 mL tubes for subsequent cell counting.
5. Count the individual cells and cell clusters of the cell suspension (Figure 2A).
  **CRITICAL:** Because there are different sizes and morphologies of exocrine cells, and many times the cell suspension will not be fully single cells, it is important to manually count the cells and cell clusters using a hemocytometer.
  a. Prepare two 0.5 mL Eppendorf tubes with 95 μL 0.02% (w/v) Trypan Blue (T8154, Sigma-Aldrich) (see materials and equipment setup section).
  b. Thoroughly resuspend cells in one 50 mL tube and pipet a 5 μL aliquot from the middle of the cell suspension to add to the Trypan Blue (20-fold dilution).
  c. Mix cells (do not vortex) in Trypan Blue and load 10 μL on the hemocytometer.
  d. Count individual cells and cell clusters.
    **CRITICAL:** Counting individual cells will give indication of the total live cells in and viability of each preparation. Based on our experience, expected cell viability is >70%. In parallel, counting clusters of cells that did not dissociate into single cells as one “cell” is important for subsequent organoid/colony assays to get the proper seeding density, as the cell cluster will not break up further upon plating in the colony assay medium. Additionally, it is recommended to record the dead cells.
  e. Repeat for cells in any additional 50 mL tubes, if applicable.
  f. Calculate the numbers and volumes of cells and cell clusters to aliquot for endpoint analyses, which may include:
    i. RNA or protein lysate collection.
    ii. Organoid/colony assay.
    iii. Cryopreservation (see step 6).

**Figure 2:**
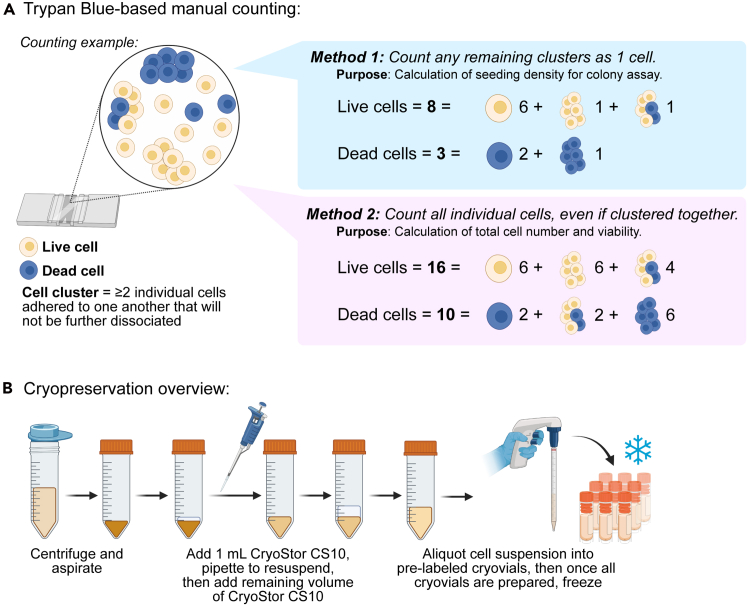
Quantification and cryopreservation of islet-depleted exocrine tissue
6. Cryopreservation of dissociated pancreatic exocrine cells (Figure 2B):
  a. Due to the variable needs in the number of cells in our two culture platforms, we cryopreserve various numbers of cells per vial (example below):

**Table:** 

No. of cells per cryovial	Cell density	Final volume per cryovial
24 x 10^6^ cells	12 x 10^6^ cells/mL	2 mL
1-10 x 10^6^ cells	1-10 x 10^6^ cells/mL	1 mL
  b. Centrifuge cells at 300–400 x g for 5 min.
  c. Aspirate supernatant and keep cells on ice (4°C).
  d. Using p1000, add 2–3 mL of CryoStor CS10 (210102, Biolife Solutions) to loosen cell pellet by gently pipette up and down a few times.
    i. Alternatively, one can use 10% (v/v) DMSO diluted in DMEM/F12 (10-092-CV, Corning) and 0.1% BSA (A8412, Sigma-Aldrich) instead of CryoStor.
  e. Prepare desired cell density by adding CryoStor and gently mix by 3–4 inversions to ensure even distribution of cell suspension.
  f. To prevent repetitive motion injury to the hand muscles, aliquot cells into cryovials using an electronic pipette, such as the PIPETBOY pro from Integra Biosciences or the Vista Lab ali-Q 2 Pipette Controller.
  g. Use a controlled rate freezer (such as the CryoMed Controlled Rate Freezer, model 7450) to minimize ice crystal formation according to the manufacturer’s protocol. See materials and equipment section for more information.
  h. Once samples are frozen, immediately transfer to a liquid nitrogen tank for long-term storage.

### Preparation of 3D suspension culture medium

**Timing: 0.5–1 h**

This step details how to prepare the suspension culture medium. Although it is recommended to prepare the medium on the day it is needed, it can be prepared up to one day in advance and stored at 4°C.

7. Prepare following reagents on ice (4°C, see “**3D suspension culture medium**” table):
  ***Note:*** The amount of reagents to thaw and media to prepare is dependent on the number of cells being thawed, the vessel the cells are cultured in, and the culture day the media is prepared. For example, for every 0.8 x 10^6^ cells thawed on day 0 we typically prepare a minimum of 1 mL of media. For the live cells collected on day 1, each 1 mL of media was used for 0.3 x 10^6^ cells.
  a. Stock solutions of growth factors: Nicotinamide, Epidermal Growth Factor (EGF), R-spondin 1 (RSPO1), Noggin, A83-01, Gastrin, and Y-27632.
  b. Knockout serum replacement (KSR, 10828-028, Thermo Fisher).
  c. DMEM/F12/PS: Dulbecco’s Modified Eagle’s Medium/Hams F-12 (10-092-CV, Corning) and 1x Penicillin-Streptomycin (15140-122, Gibco).
    ***Note:*** If using previously thawed aliquot(s) of growth factors and/or KSR, ensure the aliquot(s) has been stored at 4°C for no longer than one week.
8. Combine reagents into a 15 mL conical tube on ice according to “**3D suspension culture medium**” table:
  a. Add DMEM/F12/PS, then KSR.
  b. Using DMEM/F12/PS, make 1:100 dilutions of both Gastrin and A83-01.
  c. Add each of the growth factors into the tube with the DMEM/F12/PS and KSR.
9. Close the tube and store on ice or at 4°C for up to one day. Then, 30 min before cells are prepared, place tube into a 37°C water bath to pre-warm.

### Preparation of 3.3% (w/v) methylcellulose

**Timing: 3–5 days**

This step details how to prepare the methylcellulose reagent necessary for the 3D methylcellulose-based colony assay, which is based on a previously published step-by-step protocol.^6^

10. Prepare the following reagents and materials:
  a. Methylcellulose powder (1,500 centipoise, 9004-67-5, Shin-Etsu Chemical).
  b. 200 mL of sterile double distilled water. Store at 4°C for at least 16 h.
  c. 500 mL of 2x DMEM/F12 media (12400024, Thermo Fisher) containing 200 U/mL penicillin and 200 μg/mL streptomycin (Gibco, 15140-122). Store at 4°C for at least 16 h.
11. Remove air from double distilled water:
  a. Add 500 mL of sterile double distilled water to a 1,000 mL glass beaker covered with aluminum foil.
  b. Carefully boil for at least 30 min on a hot plate or over a Bunsen burner.
12. Dissolve methylcellulose powder in double distilled water:
  a. Place a large sized stir bar (at least 3 inches in length) into a 2000 mL glass flask.
  b. Carefully measure 300 mL of boiling water and transfer it to the flask.
  c. Place the flask on a stir plate without heating and stir the hot water slowly to avoid generating air bubbles.
  d. Weigh 11 g x 3 (total 33 g) of methylcellulose powder and very slowly add it to the hot water. Stir the powder until the temperature is reduced to approximately 40°C–45°C.
  e. Add 200 mL cold sterile double distilled water to the methylcellulose mixture.

**CRITICAL:** Immediately after adding the cold water, the solution will turn transparent and viscous, indicating that methylcellulose is dissolving into the aqueous phase. Hand swirl the flask to ensure the solution is mixed.

***Note:*** The solution will not have a smooth or even consistency at this point.

13. Add 500 mL cold 2x DMEM/F12/PS media. Continue to swirl by hand to mix.

***Note:*** The solution will not have a smooth or even consistency at this point.

14. Place the flask on a stir plate in a cold room and stir the methylcellulose solution for 2–5 days or until the viscous solution reaches a smooth consistency.
15. Aliquot the solution by pouring 100 mL volumes into 125 mL per storage bottle, and store at −20°C. Use stored aliquots within 24 months of preparation.

***Note:*** It is recommended to dispense newly prepared methylcellulose into a sterile petri dish and place in a 37°C incubator for one week to test sterility (i.e. no bacteria).

***Note:*** To thaw frozen aliquots, place at 4°C for 16–24 h. Aliquots can be stored in the fridge for up to two months.

### Preparation of 3D methylcellulose-based colony assay medium

**Timing: 0.5–1 h**

This step details how to prepare the colony assay medium just prior to adding the cells. Although it is recommended to prepare the medium on the day of cell plating, it can be prepared up to one day in advance and stored at 4°C.

16. Prepare the following reagents on ice (see materials and equipment setup section):
  a. Growth factor master mix (see “**Colony assay: Growth factor master mix**” tables).
  b. Growth factor reduced Matrigel (356231, Corning). If needed, thaw at 4°C overnight (16–24 h).
  c. DMEM/F12/PS: Dulbecco’s Modified Eagle’s Medium/Hams F-12 (10-092-CV, Corning) and 1x Penicillin-Streptomycin (15140-122, Gibco).
  d. 3.3% (w/v) Methylcellulose in DMEM/F12/PS. Thaw at 4°C for 16–24 h.
17. Using aseptic technique, prepare 5 mL polystyrene snap-cap tubes (352054, Corning) to contain the colony assay medium in a tissue culture hood (see “**Colony assay medium**” table).
  ***Note:*** Each tube will eventually contain a final volume of 2,500 μL, including cells and medium, which is sufficient to plate 1 group (or condition) of 4 wells in a 24-well plate at 500 μL/well (some volume is expected to be lost from aliquoting) (Figure 3).
  **CRITICAL:** Keep all reagents on ice, especially after Matrigel is added. Prepared medium can be stored at 4°C up to one day before use.
  a. Aliquot growth factor master mix (372.5 or 342.5 μL of the 9- or 6-factor culture, respectively) into individual polystyrene snap-cap tubes.
  b. Add 600 μL DMEM/F12/PS to each tube.
  c. Using chilled pipette tips, add 125 μL Matrigel into each polystyrene snap-cap tube. To avoid thawing of the tip, use separate chilled tips for each tube.
  d. Using a 16½ G needle (305198, BD Biosciences) mounted onto a 1 mL syringe (309659, BD Biosciences), add 750 μL of 3.3% Methylcellulose to each polystyrene snap-cap tube.
    **CRITICAL:** The use of the syringe here is necessary to accurately measure the volume of viscous methylcellulose solution. To avoid losing volume to the dead space in the needle, evacuate the air bubble after drawing in some of the solution before taking final measurement.

**Figure 3:**
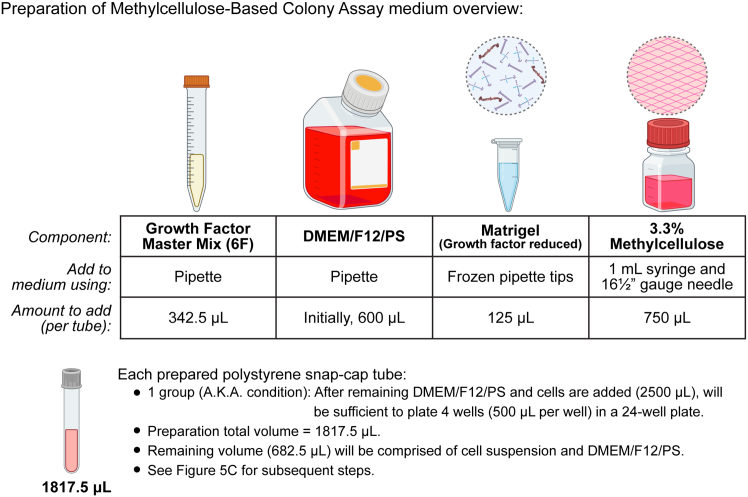
Preparation of methylcellulose-based colony assay medium
18. Snap the tube closed and store on ice or at 4°C until cells are ready for plating. Medium can be prepared up to one day in advance.

## Key resources table

**Table:** 

REAGENT or RESOURCE	SOURCE	IDENTIFIER
**Biological samples**		

Islet-depleted adult human pancreatic tissue	Southern California Islet Cell Resource Center at City of Hope	https://iidp.coh.org/Centers

**Chemicals, peptides, and recombinant proteins**		

Penicillin-streptomycin	Gibco	Cat# 15140-122
Cell recovery solution	Corning	Cat# 354253
KnockOut serum replacement (KSR)	Thermo Fisher Scientific	Cat# 10828-028
Nicotinamide	Sigma-Aldrich	Cat# N0636
Exendin 4	Sigma-Aldrich	Cat# E7144
SB-202190	Sigma-Aldrich	Cat# S7067
Gastrin II sulfated	Sigma-Aldrich	Cat# G1260
Recombinant mouse R-Spondin1 (RSPO1)	R&D Systems	Cat# 3474-RS
Recombinant human vascular endothelial growth factor (VEGF)	R&D Systems	Cat# 293-VE
Recombinant human epidermal growth factor (EGF)	R&D Systems	Cat# 236-EG
Recombinant mouse Noggin	R&D Systems	Cat# 1967-NG
A83-01	Tocris	Cat# 2939
Y-27632 (Ri)	R&D Systems	Cat# 1254/10
Recombinant human Activin B protein	R&D Systems	Cat# 659-AB
Collagenase B	Sigma-Aldrich	Cat# 11088831001
Bovine desoxyribonuclease (DNase) 1	Millipore Sigma	Cat# 260193
Bovine serum albumin (BSA) solution	Sigma-Aldrich	Cat# A8412
Fetal bovine serum	Atlanta Biologicals	Cat# S11550
Liberase TH	Sigma-Aldrich	Cat# 05 401 151 001
CTS TrypLE select enzyme	Thermo Fisher Scientific	Cat# A1285901
4′,6-diamidino-2-phenylindole (DAPI)	Invitrogen	Cat# D3571
5-ethynyl-2′-deoxyuridine (EdU)	Abcam	Cat# ab146186
Fisher Healthcare Tissue-Plus O.C.T. compound	Fisher Scientific	Cat# 23-730-571
Citrate buffer (10x)	Electron Microscopy Sciences	Cat# 64142-08
VECTASHIELD Vibrance antifade mounting medium	Vector Laboratories	Cat# H-1700-10
Vector TrueVIEW autofluorescence quenching kit	Vector Laboratories	Cat# SP-8400-15
DMEM (Dulbecco’s modified Eagle’s medium)/Hams F-12 50/50 mix	Corning	Cat# 10-092-CV
DMEM (Dulbecco’s modified Eagle’s medium)/Hams F-12 50/50 mix, powder, with HEPES	Thermo Fisher Scientific	Cat# 12400024
Dulbecco’s phosphate-buffered saline (dPBS) without calcium and magnesium	Corning	Cat# 21-031-CV
Ethylenediaminetetraacetic acid (EDTA)	Gibco	Cat# 15575-038
Paraformaldehyde solution (4%) in PBS	Santa Cruz	Cat# sc-281692; CAS 30525-89-4
Triton X-100	Sigma-Aldrich	Cat# X100-100ML
0.4% trypan blue	Sigma-Aldrich	Cat# T8154
CryoStor CS10	BioLife Solutions	Cat# 210102
Histopaque 1077	Sigma-Aldrich	Cat# 10771-100ML
Dimethyl sulfoxide (DMSO), Hybri-Max, sterile-filtered	Sigma-Aldrich	Cat# D2650

**Critical commercial assays**		

RNeasy microkit	QIAGEN	Cat #74004
TaqMan universal PCR master mix	Applied Biosystems	Cat# 4304437
QuantiTect reverse-transcription kit	QIAGEN	Cat# 205313
Click-iT EdU Alexa Fluor 488 imaging kit	Thermo Fisher Scientific	Cat# C10337

**Software and algorithms**		

ImageJ	Version 1.53e	https://imagej.nih.gov/ij/
GraphPad Prism	Version 9.5.1	https://www.graphpad.com/scientific-software/prism/
Adobe Photoshop	Version 24.4.1	https://www.adobe.com/products/photoshop.html
Adobe Illustrator	Version 27.5	https://www.adobe.com/products/illustrator.html
Zen Blue	Version 2.5	https://www.zeiss.com/microscopy/en/products/software/zeiss-zen.html

**Other**		

Matrigel growth factor reduced (GFR) basement membrane matrix, phenol red-free, LDEV-free	Corning	Cat# 356231
Methylcellulose, 1500 cPs	Shin-Etsu Chemical	CAS 9004-67-5
PIPETBOY pro electronic pipette	Integra Biosciences	Cat# 156 401
BD slip tip sterile syringes, 1 mL	BD Biosciences	Cat# 309659
BD Luer-Lock tip sterile syringes, 10 mL	BD Biosciences	Cat# 302995
BD PrecisionGlide needle 16G × 1 1/2	BD Biosciences	Cat# 305198
5 mL serological pipettes	Genesee	Cat# 12-102
10 mL serological pipettes	Genesee	Cat# 12-104
25 mL serological pipettes	Genesee	Cat# 12-106
50 mL serological pipettes	Genesee	Cat# 12-107
15 mL centrifuge tubes, bulk, sterile	Genesee	Cat# 28-103
50 mL centrifuge tubes, bulk, sterile	Genesee	Cat# 28-108
Sterile 50 mL disposable vacuum filtration system Steriflip	Fisher Scientific	Cat# SCGP00525
125 mL Corning Costar disposable storage bottles	Corning	Cat# 8388
Falcon round-bottom polystyrene tubes	Fisher Scientific	Cat# 352054
Fisherbrand sterile cell strainers, 40 μm	Fisher Scientific	Cat# 22-363-547
Fisherbrand sterile cell strainers, 100 μm	Fisher Scientific	Cat# 22-363-549
Corning Costar ultra-low attachment multiple well plate (6-well)	Corning	Cat# 3471
Corning 24-well plate, ultra-low attachment	Corning	Cat# 3473
Thermo Scientific Nunclon Sphera microplates, 96F bottom	Fisher Scientific	Cat# 174927
384-well cell culture microplates, PS, flat bottom, μClear, white, CELLSTAR, cell-repellent surface, with lid, sterile	Greiner	Cat# 781974
Corning ultra-low attachment flasks T25	Corning	Cat# 4616
Corning ultra-low attachment 75 cm^2^ U-flask	Corning	Cat# 3814
T25 CELLSTAR, cell-repellent surface, white filter screw cap, sterile	Greiner	Cat# 690985
T75 CELLSTAR cell culture flasks, cell-repellent surface, filter caps	Greiner	Cat# 658985
Seal-Rite 1.5 mL microcentrifuge tubes, natural, sterile	USA Scientific	Cat# 1615-5510
Seal-Rite 0.5 mL microcentrifuge tubes, assorted, not sterile	USA Scientific	Cat# 1605-0099
Nunclon Surface 2 × 2 mm gridded petri dish	Thermo Fisher Scientific	Cat# 169558
Epredia Peel-A-Way disposable embedding molds	Fisher Scientific	Cat# 12-20
Affi-Gel blue gel	Bio-Rad	Cat# 1537301

## Materials and equipment

CryoMed Controlled Rate Freezer (model 7450) set-up.

• Set up a profile for controlled rate freezing (the following is our protocol):
  ○ Step1: Wait at 4°C.
  ○ Step2: −1.0°C/min to −4°C.
  ○ Step3: −25.0°C/min to −40°C.
  ○ Step4: 10°C/min to −12.0°C.
  ○ Step5: −1.0°C/min to −40°C.
  ○ Step6: −10°C/min to −90°C.
  ○ End and Hold.
• If condensation is visible, dry the door, gasket, and chamber of the controlled rate freezer.
• Open liquid nitrogen valve to allow flow into the freezer.
• Replace the sentinel vial connected to the sample probe with a vial containing 1 mL fresh CryoStor CS10.
• Close the door tightly with a full turn of the handle and turn on the freezer.
• Make sure printer paper comes up through the slot.
• Using the software of the instrument, select a pre-set program, make sure it is at “step1”, and choose “Run”.
• When the sample temperature is approximately 4°C, open the door, add the cryovials into the chamber, close the door tight, and choose “Run” again.
• When the cryovials reach or are being held at −90°C (after ∼70 min), choose “End”, open the door, transfer vials to dry ice, and collect the printout.
• Close the liquid nitrogen valve, turn off the freezer, and dry any visible condensation; leave the sentinel cryovial as is and the door ajar.

Cell culture flasks and plates:

***Note:*** For both culture platforms, it is necessary to use ultra-low adherent tissue culture plates to prevent cells or colonies from adhering to the plastic. The following plates and flasks are what our laboratory typically uses:

• Ultra-low adherent plates:
  ○ 6-well: 3471, Corning
  ○ 24-well: 3473, Corning
  ○ 96-well: 174927, Thermo Scientific
  ○ 384-well: 781974, Greiner
• Ultra-low adherent flasks:
  ○ T25: 4616, Corning or 690985, Greiner
  ○ T75: 3814, Corning or 658985, Greiner

DNase1 (260193, Millipore-Sigma).

• Upon receipt, store the DNase1 powder (10 MU) at −20°C.
• To prepare 1 MU/mL stock solution, add 10 mL sterile PBS/BSA/PS and mix using gentle pipetting.
• Make 50 and 500 μL aliquots and store at −20°C. Use within 3 months of preparation.

Penicillin-Streptomycin (PS, 15140-122, Gibco).

• Solution arrives as 10,000 U/mL penicillin and 10,000 μg/mL streptomycin (100x concentration).
• Make 5.5 mL aliquots and store at −20°C. Use within 12 months of preparation.

PBS/BSA/PS: Dulbecco’s phosphate-buffered saline (PBS, 21-031-CV, Corning), 0.1% (v/v) Bovine Serum Albumin (BSA, A8412, Sigma-Aldrich), and 1x PS (15140-122, Gibco).

**Table:** 

Reagent	Stock concentration	Final concentration	Amount
PBS	N/A	N/A	500 mL
BSA	7.5%	0.1%	6.7 mL
PS	100x	1x	5 mL
**Total**	**N/A**	**N/A**	**511.7 mL**

Store at 4°C for up to 3 months.

PBS/BSA/PS/DNase1: PBS/BSA/PS and 2,000 U/mL DNase1 (260913, Millipore Sigma).

• Aliquot necessary PBS/BSA/PS.
• Add 1:500 DNase1 stock (1 MU/mL) to PBS/BSA/PS aliquot.
• Prepare on the day of use and keep on ice (4°C).

Collagenase B (11088831001, Sigma-Aldrich).

• Upon receipt, store the lyophilized powder (2.5 g) at 4°C.
• Prepare 100 mg/mL solution by adding 25 mL PBS/BSA/PS on ice. Place at 4°C overnight (14–18 h), swirling occasionally to mix.
• Once fully dissolved, filter-sterilize through a 0.2 μm filter (SCGP00525, Millipore Sigma).
• Make 1 and 6 mL aliquots and store at −20°C. Use within 6 months of preparation.

Heat-inactivated fetal bovine serum (FBS, S11550, Atlanta Biologicals).

• Upon receipt, store serum at −80°C.
• When needed, thaw at 4°C overnight (14–18 h). Once thawed, aliquot into 125 mL bottles (8388, Corning) and then place bottles at 56°C for 30 min.
• Leave bottles at 22°C for about 1 h, then store at −20°C. Use within 24 months of preparation.

Cryostor CS10 (210102, Biolife Solutions).

• Upon receipt, cover in foil and store at 4°C.
• Keep in the dark and on ice during experiment.

Matrigel Growth Factor Reduced (GFR) Basement Membrane Matrix (Matrigel, 356231, Corning).

• Upon receipt, store at −20°C.
• Thaw at 4°C for 16–24 h. Once thawed, aliquot 1 mL Matrigel into 2 mL microtubes (72.694.006, Sarstedt) using chilled p1000 pipette tips.
• Store at −20°C. Use within 12 months of preparation.

DMEM/F12/PS: Dulbecco’s Modified Eagle’s Medium/Hams F-12 (DMEM/F12, 10-092-CV, Corning) and 1x PS (15140-122, Gibco).

• Take one 500 mL bottle of DMEM/F12 from 4°C storage and open in tissue culture hood using aseptic technique.
• Thaw PS aliquot and add 5 mL to DMEM/F12 bottle (1x final concentration).
• Store at 4°C. Use within 3 months of preparation.

**Table:** DMEM/F12/BSA/PS: DMEM/F12/PS, 0.1% (v/v) BSA (A8412, Sigma-Aldrich), 1x PS (15140-122, Gibco)

Reagent	Stock concentration	Final concentration	Amount
DMEM/F12	N/A	N/A	500 mL
BSA	7.5%	0.1%	6.7 mL
PS	100x	1x	5 mL
**Total**	**N/A**	**N/A**	**511.7 mL**

Store at 4°C for up to 3 months.

5-ethynyl-2′-deoxyuridine (EdU) (ab146186, Abcam).

• Upon receipt, store at −20°C in desiccator.
• Weigh 2.522 mg and reconstitute in 1 mL PBS (10 mM stock concentration).
• Store at 4°C and use within one week. Alternatively, store at −20°C and use within 1 month of preparation.

Liberase Thermolysin High (Liberase TH, 05 401 151 001, Sigma-Aldrich).

• Upon receipt, store at −20°C.
• To reconstitute to 0.56 U/mL final concentration, suspend 50 mg Liberase TH in 464 mL of DMEM/F12.
• Make 5 and 50 mL aliquots and store at −80°C. Use within 3 months of preparation.

Sucrose (117140010, Thermo Scientific): 30% (w/v) sucrose solution.

• Weigh 9 g sucrose and add to a 50 mL conical tube.
• Add 21 mL sterile dH_2_O and invert to mix.
• Filter-sterilize through 0.2 μm filter (SCGP00525, Millipore Sigma) and store at 4°C. Use within 3 months of preparation.

PBS/EDTA: PBS (21-031-CV, Corning) and 5 mM Ethylenediaminetetraacetic acid (EDTA).

• Aliquot 9.9 mL of PBS in tissue culture hood using aseptic technique.
• Add 100 μL EDTA (stock 0.5 M, 15575-038, Gibco) to PBS to get 5 mM final concentration.
• Keep on ice or store at 4°C. Use within two weeks of preparation.

Trypan Blue: 0.02% (w/v) Trypan Blue solution.

• Add 2.5 mL stock (0.4%) Trypan Blue (T8154, Sigma-Aldrich) to 47.5 mL PBS (21-031-CV, Corning).
• Filter-sterilize through 0.2 μm filter (SCGP00525, Millipore Sigma) and store at 22°C.

Paraformaldehyde and Triton X-100 (PFA/TX100): 4% Paraformaldehyde (PFA, stock 4% solution, sc-281692, Santa Cruz Biotechnologies) and 0.01% Triton X-100.

• Upon receipt, make 5 and 10 mL aliquots of 4% PFA solution and store at −20°C. Use within 12 months of preparation.
• Thaw and store aliquots at 4°C and use within one week of thawing.
• If needed for fixation, add 0.01% (v/v) of Triton X-100 (TX100, stock 100%, X100-100ML, Sigma-Aldrich). TX100 addition to PFA helps to prevent spheroids and colonies from sticking to conical or Eppendorf tubes.

**Table:** 3D suspension culture medium

Reagent	Stock concentration	Final concentration	Volume
Serum replacement	100%	10% volume	100 μL
Nicotinamide	1 M	10 mM	10 μL
EGF	25 μg/mL	50 ng/mL	2 μL
Noggin	10 μg/mL	100 ng/mL	10 μL
A83-01∗	50 mM∗	500 nM	1 μL
Gastrin II Sulfated∗	1 mM∗	100 nM	10 μL
R-Spondin 1	250 μg/mL	0.75 μg/mL	3 μL
Y-27632∗∗	10 mM	10 μM	1 μL
DMEM/F12/PS	N/A	N/A	0.863 mL
**Total volume**	**N/A**	**N/A**	**Per 1 mL**

Store at 4°C for up to one day before use. At least 10 min prior to usage, warm in 37°C water bath.

∗Make 1:100 dilutions of these reagents before adding to master mix.

∗∗Y-27632 is only included from day 0 until day 1.

**Table:** Colony assay: Growth factor master mix (9 factor medium)

Reagent	Stock concentration	Final concentration	Volume
Knockout serum replacement	100%	10%	250 μL
Nicotinamide	1 M	10 mM	25 μL
Exendin 4	0.1 μM	0.1 nM	2.5 μL
VEGF	10 μg/mL	10 ng/mL	2.5 μL
EGF	25 μg/mL	50 ng/mL	5 μL
Noggin	10 μg/mL	100 ng/mL	25 μL
A83-01∗	50 mM	500 nM	2.5 μL
SB-202190∗	100 mM	10 μM	25 μL
Gastrin II Sulfated∗	1 mM	100 nM	25 μL
R-Spondin 1	250 μg/mL	0.75 μg/mL	7.5 μL
Y-27632	10 mM	10 μM	2.5 μL
**Total**	**N/A**	**N/A**	**372.5 μL**

Store at 4°C for up to one day before use.

∗Make 1:100 dilutions of these reagents before adding to master mix.

**Table:** Colony assay: Growth factor master mix (6 factor medium)

Reagent	Stock concentration	Final concentration	Volume
Knockout serum replacement	100%	10%	250 μL
Nicotinamide	1 M	10 mM	25 μL
EGF	25 μg/mL	50 ng/mL	5 μL
Noggin	10 μg/mL	100 ng/mL	25 μL
A83-01∗	50 mM	500 nM	2.5 μL
Gastrin II Sulfated∗	1 mM	100 nM	25 μL
R-Spondin 1	250 μg/mL	0.75 μg/mL	7.5 μL
Y-27632	10 mM	10 μM	2.5 μL
**Total**	**N/A**	**N/A**	**342.5 μL**

∗Make 1:100 dilutions of these reagents before adding to master mix.

**Table:** Colony assay medium

Reagent	Final concentration	9 factor medium	6 factor medium
Growth factor master mix	N/A	372.5 μL	342.5 μL
DMEM/F12/PS∗ + cell suspension	N/A	1252.5 μL	1282.5 μL
Matrigel (8-12 mg/mL)	5% v/v	125 μL	125 μL
Methylcellulose (3.3% in DMEM/F12)	1% w/v	750 μL	750 μL
**Total**	**N/A**	**2500 μL**	**2500 μL**

Store at 4°C for up to one day before use.

∗DMEM/F12/PS volume subject to change depending on cell suspension volume.

## Step-by-step method details

### 3D suspension cell culture: Ductal cell spheroid formation

**Timing: 7 days for all steps**

**Timing: 1 h for step 1**

**Timing: 2 h for step 2**

**Timing: 0.5–1 h for step 3**

**Timing: 0.5 h for step 4**

**Figure 4:**
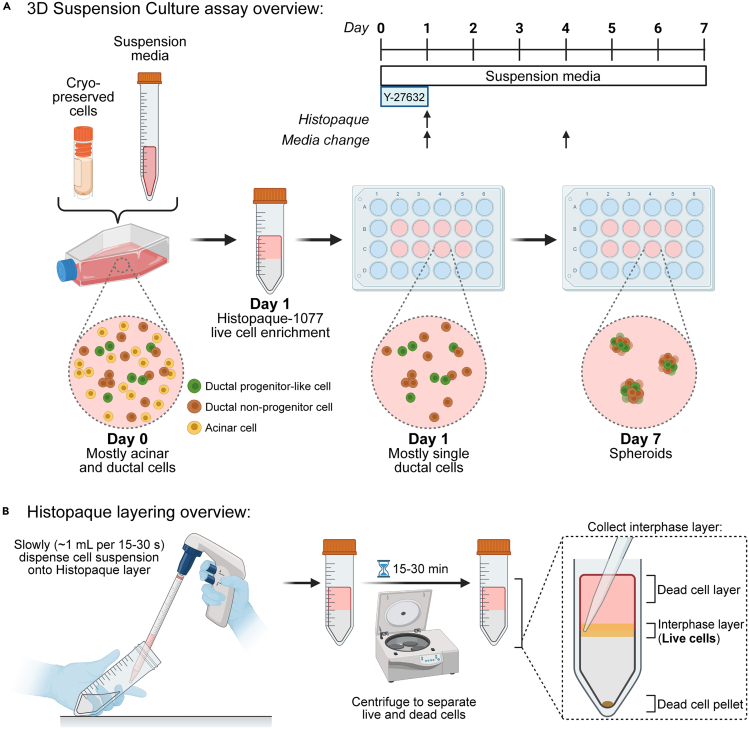
3D suspension culture of cryopreserved exocrine cells derived from adult human pancreas This culture method allows the preferential survival of ductal not acinar cells.

This section describes the protocol for generating ductal cell spheroids from primary human exocrine cells in our 3D suspension cell culture platform. A sub-population in the spheroids contains ductal cells with progenitor cell capacity.^1^ Refer to Figure 4A for general experimental overview.

1. Day 0: Thaw cryopreserved exocrine cells.
  a. In 37°C water bath, pre-warm PBS/BSA/PS/DNase1 (see materials and equipment setup section for more information):
    i. For each 1 mL of cryopreserved cells, prepare 10 mL of PBS/BSA/PS/DNase1.
  b. In 37°C water bath, pre-warm 3D suspension culture medium (see “Preparation of 3D Suspension Culture Medium” section above):
    i. For 24-well plate, prepare 0.5 mL suspension culture medium per 0.4 x 10^6^ cells per well.
    ii. For T25 flask, prepare 6–7 mL suspension culture medium per 10-12 x 10^6^ cells per flask.
  c. Retrieve cryovial(s) from liquid nitrogen tank and keep on dry ice.
  d. Place cryovial(s) into 37°C water bath for about 2 min, or until only a small chunk of ice is visible.
  e. Spray cryovial(s) with 70% EtOH to sterilize and transfer to a tissue culture hood.
  f. Using aseptic technique, gently transfer cells into a 15 mL conical tube.
  g. Pipette 1 mL PBS/BSA/PS/DNase1 into cryovial to get any remaining cells and add to the conical tube.
  h. Gradually add warm PBS/BSA/PS/DNase1 to the conical tube while gently swirling up to 10 mL final volume (can take ∼1-2 min).
    **CRITICAL:** We recommend to pre-warm the PBS/BSA/PS/DNase1 in a 37°C water bath before adding to cells. We tested in parallel whether 37°C versus 22°C PBS/BSA/PS/DNase1 impacted cryopreserved human exocrine cell survival, and found that the use of 37°C warmed solution better supports the survival of cells in 3D suspension culture.
  i. Centrifuge at 300–400 × *g* for 5 min.
    ***Note:*** All centrifugation steps for live cells conducted in this protocol should be done at ambient (∼22°C) temperature.
  j. Aspirate supernatant and resuspend cell pellets gently in pre-warmed suspension culture medium using p1000.
  k. Add enough medium for plating into ultra-low adherent culture plate or flask.
  l. Place cells in incubator (5% CO_2_, 37°C) until the next day.
2. Day 1: After overnight incubation, enrich live cells using Histopaque 1077 (10771-100ML, Sigma-Aldrich):
  a. Prepare Histopaque by allowing to come to 22°C:
    i. For handling up to 5 x 10^6^ cells, add 3 mL of Histopaque into a 15 mL conical tube.
    ii. For handling up to 25 x 10^6^ cells, add 15 mL of Histopaque into a 50 mL conical tube.
  b. Pre-warm DMEM/F12/BSA/PS in 37°C water bath.
  c. Obtain the 24-well plate(s) or T25 flask(s) of cells and transfer into a separate, empty 15 (or 50) mL conical tube.
  d. Wash the remaining cells in well(s) or flask(s) with 0.5–1 mL warm DMEM/F12/BSA/PS and add to conical tubes.
  e. Fill conical tubes up to 14 (or 20) mL with warm DMEM/F12/BSA/PS.
  f. Spin down cells at 300 x g for 5 min.
  g. Aspirate supernatant and resuspend cells gently in 3 (or 15) mL DMEM/F12/BSA/PS, depending on cell number.
  h. Very slowly layer 3 (or 15) mL of cell suspension into the tube containing 3 (or 15) mL of Histopaque (Figure 4B).
    **CRITICAL:** To maintain the interface between Histopaque and cell suspension, add the cells very slowly. It is recommended to use an electronic pipette controller with the speed setting at the slowest rate, and to hold both the tube and pipette at a 45° angle. Each mL of cell suspension can take around 15–30 s to dispense.
  i. Centrifuge the tubes at 400 x g for 15–30 min and ensure that the acceleration and deceleration settings are set at the lowest level.
    ***Note:*** 15 mL tubes can be centrifuged for 15 min, 50 mL tubes for 30 min. The total time for the centrifugation using these settings is around 30–50 min.
    ***Note:*** The settings used on a Sorvall ST40R centrifuge: 400 x g, 15 (or 30) min, 20°C, Acc 1, Dec 0.
  j. During Histopaque centrifugation, prepare 3D suspension culture medium (without Y-27632), and pre-warm in a 37°C water bath unless otherwise stated:
    i. For 24-well plate, add 0.5 mL suspension medium per 1.5 x 10^5^ cells per well.
    ii. For T25 flask, add 6–7 mL suspension medium per 5–6 x 10^6^ cells per flask.
      ***Note:*** If treating suspension cells with ECM proteins, such as Matrigel, then prepare 3D suspension culture medium on ice. Then, 10 min before the cells are prepared for plating, add the ECM proteins, mix by inversion, and allow to come to 22°C.
  k. Once the Histopaque cell separation is complete, carefully transfer the tube(s) to the cell culture hood without disturbing the liquid layers.
  l. Collect 1–2 (or 4–5) mL of the interface layer (thin cloudy cell layer) using a p1000 and transfer cells into a new 15 (or 50) mL conical tube (Figure 4B). See problem 3 if many dead cells or cell fragments are collected at this step.
  m. Bring volumes up to 14 (or 20) mL with warm DMEM/F12/BSA/PS and centrifuge cells at 300–400 x g for 5 min with standard acceleration and deceleration settings.
  n. Wash cells once more, and aspirate and discard the supernatant.
  o. Resuspend pellets in 1 mL 3D suspension culture media, then take small aliquots (10–30 μL) to count cells using a hemocytometer.
    ***Optional:*** During counting, cells can be stored with a loosened cap in incubator (37°C, 5% CO_2_) for up to 1 h.
    ***Note:*** By day 1, it is expected that most of the cells will be single cells. However, some cells adhere to one another in a cell cluster, and the amount of clustering can vary from sample to sample. Thus, to normalize the number of cells plated on day 1, count individual cells at this step instead of the cell clusters if plating the cells into suspension culture.
  p. Once counting is completed, calculate any dilutions needed to obtain the appropriate cell concentration in 3D suspension culture medium (see above step 2j).
  q. Once the cells are diluted to the appropriate concentration, plate cells in new ultra-low adherent culture plate(s) or flask(s).
  r. Place cells into incubator (37°C, 5% CO_2_) until day 4.
3. Day 4: Replenish growth factors in suspension culture.
  a. Prepare and pre-warm 3D suspension culture medium (without Y-27632) in 37°C water bath.
  b. Using gentle pipetting, collect wells or flasks of cells into Eppendorf tubes or conical tubes.
  c. Centrifuge at 100–150 x g for 1 min.
    **CRITICAL:** One may need to optimize the timing of centrifugation such that cells are brought to the bottom of the tube without compromising their morphology, such as shearing the cystic structures in 5% Matrigel culture conditions. To ensure morphology is retained, visualize the cells, spheroids, or cysts in a light microscope before and after centrifugation to see if there are any significant changes. One example of a modification our laboratory made to bring cells cultured in the presence of 5% Matrigel to the bottom of the tube was to centrifuge for 3 min at 150 x g instead of 1 min.
  d. Slowly aspirate and remove around 70–80% supernatant.
    ***Note:*** It is recommended to use a p200, and to examine the removed supernatant using a microscope to ensure minimal live cells, spheroids, or cysts were aspirated.
    ***Optional:*** If significant numbers of cells/spheroids/cysts were aspirated, then centrifuge the collected supernatant at 400 x g for 5 min, aspirate the supernatant carefully, then pool any pelleted cells with the original tube of cells.
  e. Replenish aspirated old medium with new warm medium, gently pipetting to disrupt pellet and resuspend cells.
  f. Re-plate cells and place in incubator until termination of experiment on day 7.
    ***Optional:*** If extending the experiment past day 7, replace the medium using the same method (steps 3a-3f) every 3 days.
4. Optional Day 6: Add thymidine analog EdU for S phase labeling.
  a. Prepare or thaw EdU stock aliquot and keep on ice (see materials and equipment setup section).
  b. From the EdU stock (10 mM), make dilution to 110 μM using DMEM/F12/PS.
  c. Add 50 μL of the 110 μM dilution to each well containing cells to get a final volume of 550 μL (if using a 24-well plate); the final concentration of EdU is 10 μM.
  d. Place the plate back into the incubator (37°C, 5% CO_2_) for up to 24 h.

***Note:*** Our laboratory has tested 24 h EdU incubation to capture actively proliferating cells (EdU^+^KI67^+^) and cells that are EdU^+^KI67^-^, which indicates a cell that has undergone at least one round of replication and then has exited the cell cycle (either in G0 or early G1).^1^^,^^7^

### Frozen embedding of suspension cultured ductal spheroids for immunofluorescence staining

**Timing: 3 days**

**Figure 5:**
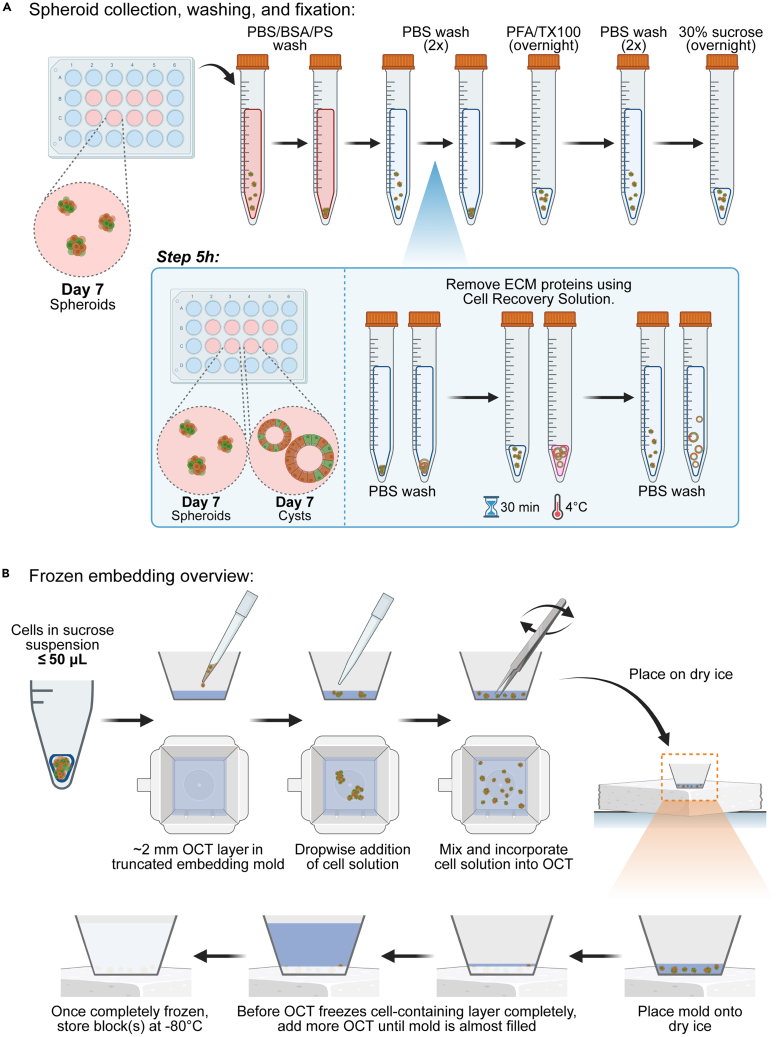
Processing of suspension cultured ductal cells for frozen embedding

This section details methods to isolate, wash, fix, and frozen embed the suspension cultured spheroids for subsequent immunofluorescence staining and analysis. Refer to Figure 5 for experimental overview.

5. Fixation of spheroids using paraformaldehyde (PFA, sc-281692, Santa Cruz Biotechnology) and Triton X-100 (TX100, Sigma-Aldrich, X100-100ML) (Figure 5A):
  a. Prepare reagents (see materials and equipment setup section for more information):
    i. PBS/BSA/PS: Pre-warm in 37°C water bath.
    ii. PBS: Keep at 22°C.
    iii. PFA/TX100: Keep on ice (4°C).
    iv. Cell Recovery Solution (only if culturing cells with 5% Matrigel or other ECM proteins): Keep on ice (4°C).
  b. Collect spheroids into a 15 mL conical tube. Rinse each well with 1 mL PBS/BSA/PS.
    ***Note:*** It is recommended to image the spheroids grown in plate prior to collection using a light microscope mounted with a camera (such as the Lumenera INFINITY 2-1R Microscopy camera, Edmund Optics).
  c. Fill up to 10 mL with warm PBS/BSA/PS and invert tubes to mix well.
  d. Spin cells down at 300 x g for 5 min.
    ***Note:*** Because keeping morphology intact for immunostaining is important for 5% Matrigel-treated cysts, we adjust centrifugation time to 2–3 min, instead of 5 min.
  e. Remove supernatant carefully, using p200 if needed.
  f. Resuspend the pellet of cells in 1 mL of PBS, then fill up to 10 mL with PBS.
  g. Centrifuge and remove supernatant carefully.
  h. If cells were cultured with ECM proteins (such as 5% Matrigel), resuspend the pellet with 1 mL Cell Recovery solution (Corning, 354253); otherwise, resuspend cells in PBS. Incubate at 4°C for 30 min, flicking the tube gently every 10 min.
    ***Note:*** Only do this step (5h) if one of the culture conditions contains ECM proteins. If additional troubleshooting is needed to remove ECM proteins, see problem 4.
  i. Centrifuge and remove supernatant carefully.
  j. Resuspend the pellet with 1 mL PBS.
  k. Centrifuge and remove supernatant carefully.
  l. Resuspend the pellet with 1 mL of 4% PFA.
  m. Incubate for 14–18 h at 4°C.
6. Sucrose incubation of spheroids:
  a. Prepare 30% sucrose solution (see materials and equipment setup section) and PBS and keep on ice (4°C).
  b. Make sure spheroids are at bottom of tube. If they are not, centrifuge at 300 x g for 1–3 min.
  c. Remove PFA/TX100 using a p200 and wash the spheroids with 1 mL PBS.
  d. To preserve the spheroid or cyst morphology, let cells sit on ice (4°C) for 10 min to allow them to sink by gravity.
  e. Remove as much PBS as possible using a p200 and wash once more with 1 mL PBS.
  f. Let cells sit on ice (4°C) for 10 min to allow them to sink by gravity.
  g. Remove as much PBS as possible and add 0.5–1 mL of 30% sucrose.
  h. Incubate for 14–18 h at 4°C.
7. Frozen embedding of spheroids in optimal cutting temperature (OCT) compound (23-730-571, Fisher Scientific) (Figure 5B):
  ***Note:*** Spheroids/cysts should be translucent.
  a. Obtain a block of dry ice with a flat surface.
  b. Obtain and label 12 × 12 × 20 mm truncated embedding molds (12–20, Fisher Scientific).
    ***Optional:*** If cells have not sunk to the bottom of the tube, centrifuge at 300 x g for 1-3 min.
  c. Using a p200, remove as much 30% sucrose as possible.
    ***Note:*** In order to not dilute the OCT, which may make cryosectioning difficult, it is recommended to have less than 50 μL 30% sucrose remaining. See problem 5 if having difficulty removing sucrose.
  d. Cut a p200 tip by approximately 0.5–1 cm using razor blade. Angle cut if wider bore is needed.
  e. Add OCT to coat the bottom of the truncated mold (∼2 mm layer of OCT, up to 5 mm in thickness), then using the cut p200 tip, gently pipette drops of cells on top of OCT layer.
  f. Use clean, sharp-tipped tweezers to mix cells into OCT.
    ***Note:*** It is recommended to do this under a dissection microscope to visualize the spheroids and sucrose. Make sure that sucrose ribbons disappear, and cells are well mixed. If bubbles form, they can be pushed to the side or given 5 min to rise.
    ***Optional:*** Add blue beads (1537301, Bio-Rad) to the edge of the OCT or mark the side of the mold to indicate where the thin, cell-containing layer stops.
  g. Freeze by placing the truncated mold directly onto dry ice. Once ice rises almost to the top of the thin layer (∼1 min), add more OCT until it is almost full.
    **CRITICAL:** Do not allow the thin layer to fully freeze before adding more OCT, as this can create a weaker bond that may chip off during subsequent cryosectioning.
  h. Once the block has fully frozen (∼15 min), store immediately at −80°C.

### 3D methylcellulose-based colony assay: Ductal progenitor-like cell analysis

**Timing: 3 weeks for all steps**

**Timing: 0.5–1 h for step 10**

**Timing: 0.5–1 h for step 11**

**Figure 6:**
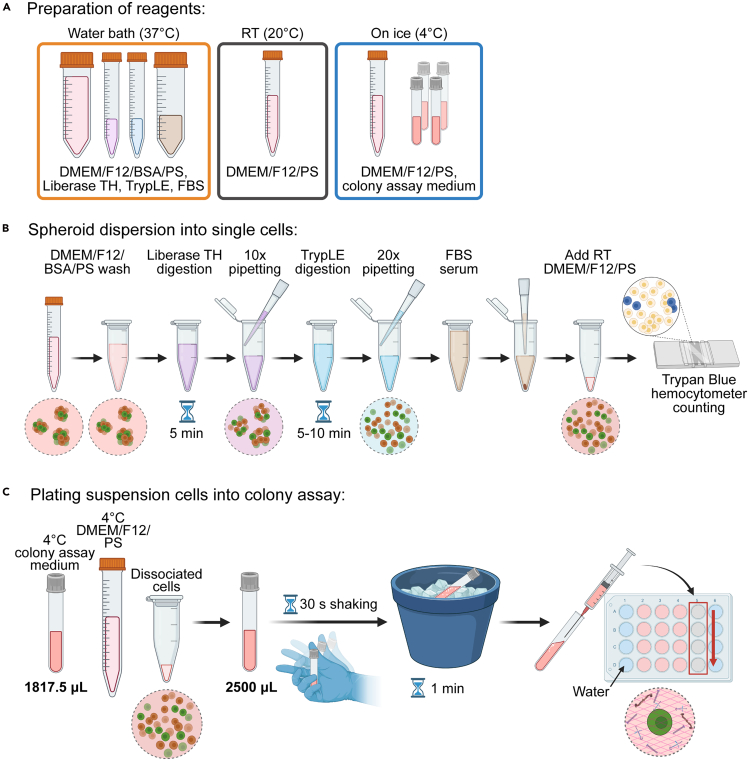
Dissociation and re-plating of suspension cultured ductal cells into methylcellulose-based colony assay

This section details the dispersion and replating of suspension cultured spheroids into a methylcellulose-based colony assay. The colony assay allows quantification of various biological functions of colony-forming progenitor cells, such as survival, self-renewal, and differentiation.^1^^,^^2^ Refer to Figure 6 for experimental overview.

8. Prepare the following reagents or aliquots (see materials and equipment setup section for more information) (Figure 6A):
  a. Pre-warm in 37°C water bath: DMEM/F12/BSA/PS, Liberase TH (05 401 151 001, Sigma-Aldrich), TrypLE Select Enzyme (A1285901, Thermo Fisher), and heat-inactivated FBS.
  b. 22°C: DMEM/F12/PS.
  c. 4°C: DMEM/F12/PS, methylcellulose-based colony assay medium snap-cap tube(s) (described in “Before you begin: Preparation of 3D Methylcellulose-based Colony Assay Medium”).
9. Prepare 24-well plates by adding sterile water into unused wells along the edges of the plate and in between each well.

***Note:*** This step is to minimize evaporation of the colony assay medium during the three-week incubation period.

10. Dissociation of suspension cultured cells (Figure 6B):
  ***Note:*** If using day 1 cells, there is typically no need for dissociation. Observe a sample of the cells under a light microscope to determine whether the grouped cells can be quantified using a hemocytometer.
  a. Collect cells into an Eppendorf or conical tube, washing each well or flask with 0.5–1 mL DMEM/F12/BSA/PS.
  b. Fill each Eppendorf up to 1.3 mL or each conical up to 10 mL with DMEM/F12/BSA/PS.
  c. Centrifuge cells at 300 x g for 5 min.
  d. Aspirate supernatant and wash once more with DMEM/F12/BSA/PS.
  e. Centrifuge spheroids at 300 x g for 5 min. If needed, transfer cells into 1.5 mL Eppendorf tubes.
    ***Note:*** It is recommended that each Eppendorf tube contains up to 200,000 live cells. If it is expected that the tube contains more than this amount, split into different Eppendorf tubes to allow adequate digestion.
  f. Remove supernatant and add 0.5–1 mL warmed Liberase TH (0.56 U/mL) and incubate at 37°C for 5 min in a 37°C water bath.
    ***Note:*** If expecting about 50,000 live cells or fewer, it is recommended to use 0.5 mL Liberase TH. For counts greater than 50,000 cells or if dissociating more than a single 24-well well of cells, use up to 1 mL.
  g. Pipette cells along the tube wall ten times using p1000 to disrupt spheroids/cysts.
  h. Centrifuge at 400 x g for 5 min.
  i. Remove supernatant, flick to disrupt the pellet, and add 0.5–1 mL warmed (undiluted) TrypLE.
    ***Note:*** If expecting about 50,000 live cells or fewer, it is recommended to use 0.5 mL TrypLE. For counts greater than 50,000 cells or if dissociating more than a single 24-well well of cells, use up to 1 mL.
  j. Incubate at 37°C for 5 min in a water bath. See problem 6 if need to optimize digestion steps.
  k. Pipette cells along the tube wall twenty times using a p1000.
    ***Note:*** It is recommended to check the dissociation after pipetting 20 times to ensure that around 80–90% of cells are single. If many spheroids or large clusters are still present, return tubes to 37°C water bath for 30–60 s and pipette another 10 times.
    **CRITICAL:** Cells can be exposed to TrypLE for up to 10 min before significant cell death.
  l. Add at least 10% (v/v) warm FBS to stop TrypLE reaction and invert tube to mix well.
  m. Centrifuge at 400 x g for 5 min.
11. Quantify live cells and plate in methylcellulose-based colony assay medium.
  a. Resuspend cell pellets with DMEM/F12/PS (22°C).
  b. Count cells and cell clusters using 0.02% Trypan blue and a hemocytometer.
    ***Note:*** For calculating seeding density, any remaining cell clusters with at least one live cell will be counted as a single live cell. It is recommended to count both the cell clusters (to calculate the colony assay seeding density), as well as individual cells (to calculate the total cells and cell viability) (Figure 2A).
    ***Optional:*** During counting, cells can be stored with a loosened cap in incubator (37°C, 5% CO_2_) for up to 1 h.
  c. Calculate the amount of cell suspension and cold DMEM/F12/PS needed to add to each prepared polystyrene snap-cap tube.
    ***Note:*** The remaining volume to make a final volume of 2,500 μL should be 682.5 μL.
  d. Add cold DMEM/F12/PS followed by cells to each prepared snap-cap tube with the colony assay medium (Figure 6C).
  e. Snap the tubes closed and shake vigorously for around 30 s.
    ***Note:*** It is recommended to handle up to four tubes at once, shaking up to two tubes securely in each hand.
  f. Using a 1 mL syringe mounted with a 16½ gauge needle, transfer 0.5 mL per well of the medium to four wells in a 24-well plate.
    ***Note:*** For each tube (4 wells), it is recommended to add the medium sequentially from the top to the bottom of one column (e.g. column 2: A, B, C, then D). Additionally, avoid any remaining bubbles.
  g. Once finished, place the plate into an incubator (37°C, 5% CO_2_) for three weeks.

### Processing of colonies and fixation for immunostaining

**Timing: 3 days**

**Figure 7:**
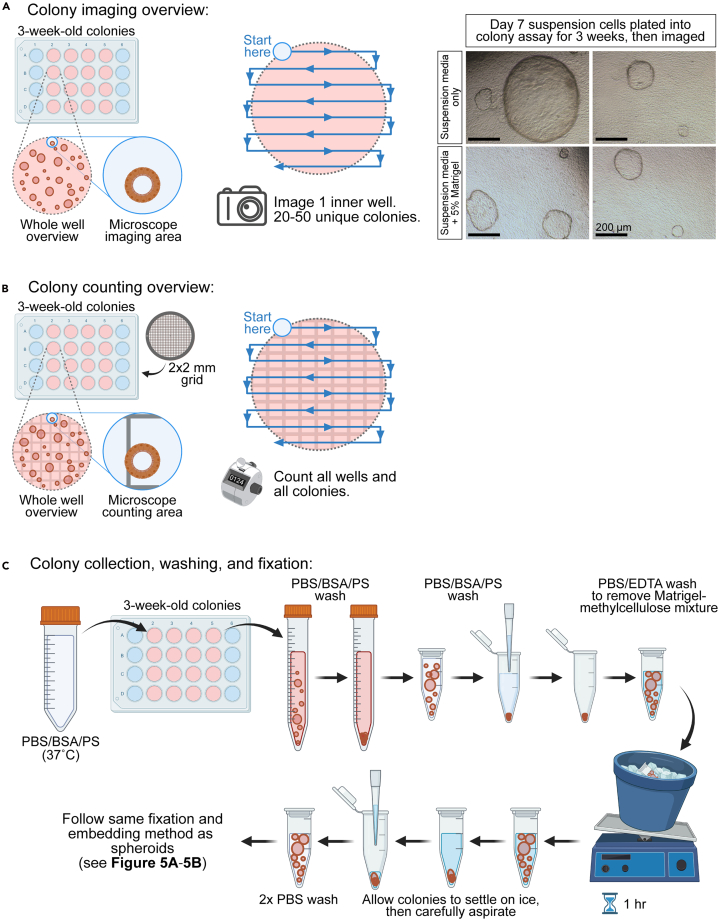
Analysis and processing of 3-week-old colonies Colonies with a cystic structure can be observed 2-3 weeks after dissociated suspension cells are plated into the colony assay. Scale bars: 200 μm.

This section describes how to quantify the number and size of colonies, as well as how to process colonies for fixation and subsequent frozen embedding for immunofluorescence analysis. In general, human pancreatic ductal colonies complete their growth in size around day 19–21 in our colony assay.^2^ Refer to Figure 7 for experimental overview, and to troubleshoot some potential problems with colony growth, see problem 7.

12. Quantification of colony diameters (Figure 7A):
  a. Using a light microscope mounted with a camera, image 20–50 colonies per group (or condition).
    ***Note:*** Colonies are counted if they are at least 50 μm in diameter. Our laboratory typically uses a 4x lens on the Olympus CKX41 inverted microscope to image colonies.
    i. Collect colony images in an unbiased manner by finding the first colony at the top left of the well, then proceeding towards the right, collecting images of all colonies along the way.
    ii. Proceed in a snake-like pattern down the well until up to 50 unique colony images are collected.
  b. Using imaging software (such as ImageJ or Adobe Photoshop), measure the diameters of 20-50 unique colonies per group.
    ***Note:*** Colonies are not perfectly circular, so one can measure the long and short diameter and then take the average. Alternatively, one can use software that measures area or circumference in place of diameter when quantifying colony size.
13. Quantification of colony-forming efficiency (Figure 7B):
  a. Using a light microscope, count all colonies in each well per group. For significant differences in colony counts in technical replicates of the same condition, refer to problem 8**.**
    ***Note:*** To assist with counting, it is recommended to use a plastic grid and to use an objective where the top grid line and bottom grid line align with the top and bottom of the field of view. This way, when moving from the top left to right, all colonies counted inside of the grid lines will be unique from those counted in the next row down.
    ***Note:*** Our laboratory uses a grid derived from the Nunclon Surface 2 × 2 mm gridded petri dish (169558, Thermo Fisher). We visualize the colonies on an Olympus CKX31 inverted microscope using the 10x objective lens.
  b. Calculate the colony-forming efficiency by dividing the colony counts by the number of cells seeded per well (seeding density, which included cell clusters).
    ***Note:*** The colony-forming efficiency is a ratio of colony-forming progenitor-like cells to total cells plated and will result in a percentage value.
    i. Calculate the colony-forming efficiency for each individual well to determine variation among technical replicates.
    ii. Calculate the average colony-forming efficiency per group to compare different groups.
14. Colony collection and fixation (Figure 7C):
  a. Prepare reagents (see materials and equipment setup section for details):
    i. PBS/BSA/PS: Pre-warm in a 37°C water bath.
    ii. PBS: Keep at 22°C.
    iii. PBS/EDTA and PFA/TX100: Keep on ice (4°C).
  b. Add 0.5 mL warm PBS/BSA/PS to each well containing colonies, swirl gently with pipette tip, and place the mixture into a 15 mL conical tube.
    ***Note:*** It is recommended to collect only up to four wells of colonies per conical tube.
  c. Wash each well with 1 mL PBS/BSA/PS and add to the conical tube.
  d. Fill up to 10 mL, then centrifuge at 400 x g for 5 min.
  e. Aspirate supernatant and resuspend in 1 mL PBS (22°C), then transfer to a 1.5 mL Eppendorf tube.
  f. Centrifuge at 400 x g for 5 min.
  g. Aspirate supernatant and resuspend in 1 mL cold PBS/EDTA.
  h. Place tubes horizontally on ice (4°C) and place on a rotator, rocker, or shaker set at a low speed for 1 h. During this incubation, place PBS on ice or at 4°C to cool.
    ***Note:*** Gentle agitation is recommended for the colonies to maintain their morphology with minimal tearing. For example, we set a rocker to a speed so that the colonies roll to each side once every ∼5 s to minimize tearing. For troubleshooting ECM protein and methylcellulose removal, see problem 9.
  i. After the incubation, place tubes vertically on ice (4°C) and allow gravity to pull colonies down for about 10 min.
    ***Note:*** If it is necessary to maximize retention of colonies for fixation, then collect the supernatant into a separate Eppendorf tube and centrifuge at 150-300 x g for 1–3 min to pellet any lost colonies. Then gently resuspend them and pool with the original tube containing the fixed colonies of the same condition.
  j. Aspirate slowly using p200, then add 1 mL cold PBS and allow gravity to pull colonies down for about 10 min.
  k. Repeat cold PBS wash once more, aspirate using p200, then add 0.5–1 mL PFA/TX100.
  l. Place tubes at 4°C for 14–18 h.
  m. Continue with frozen embedding protocol as indicated for suspension cultured spheroids/cysts in steps 6-7.

## Expected outcomes

**Figure 8:**
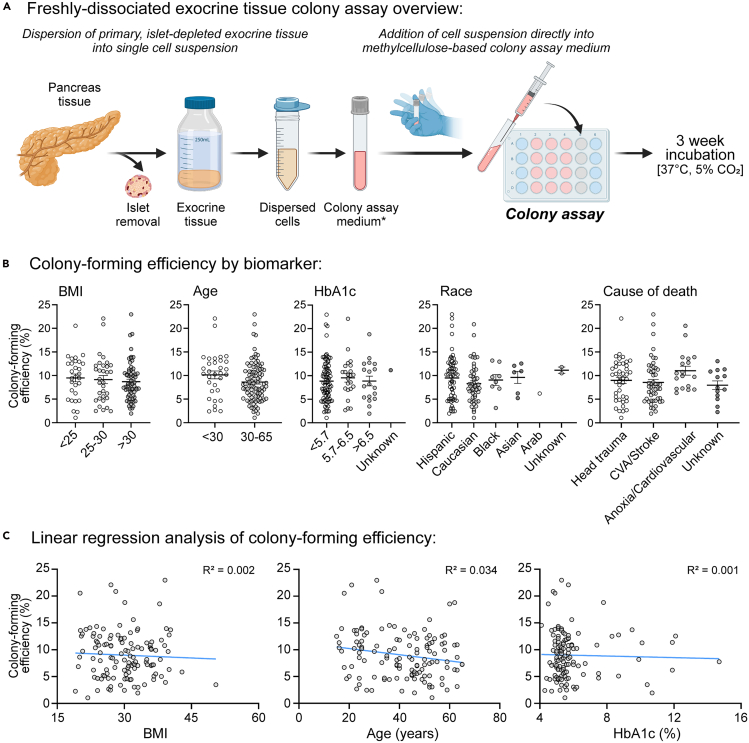
Analysis of colony-forming efficiency from human exocrine cells Our laboratory routinely examines whether freshly dissociated exocrine cells (without cryopreservation) from each cadaveric donors can form colonies. Data below are derived from a total of 124 donors; colonies are formed from all donors tested to date. See Table 1 for the summary of donor characteristics. (A) Overview of methylcellulose-based colony assay procedure using freshly dissociated (or non-cryopreserved) exocrine cells. ∗Indicates that the colony assay medium contained VEGF, SB-202190, Exendin 4, and Activin B (see Quijano et al. 2023 for details).^2^ (B) Analysis of colony-forming efficiency (calculated as number of 3-week-old colonies divided by the number of cells plated at day 0) based on the following recorded biomarkers: body-mass index (BMI), age, hemoglobin A1c (HbA1c), race, and cause of death. Data are depicted as mean ± SEM, and each dot represents a unique donor. All data were analyzed by one-way ANOVA, except for the age analysis, with which Welch’s two-tailed t-test was used. No statistical significance was found. (C) Linear regression analysis of colony-forming efficiency based on BMI, age, and HbA1c. No correlation was found.

Here, we describe methods to isolate and culture primary ductal cells derived from cadaveric, islet-depleted pancreatic exocrine tissues. Thus far, our laboratory has tested 124 individual donor exocrine tissues (Table 1), and all donor tissues have yielded colonies in the methylcellulose-based colony assay (Figure 8). In addition to cryopreservation of exocrine cells from donors without apparent diseases, we also collected cells from donors with type 2 diabetes, which allows for future studies to examine the impact of disease on the ductal cells.

**Table 1:** Characteristics of cadaveric human pancreas donors examined

Tissue biomarkers		Number of donors	Colony-forming efficiency (mean ± SEM)
Sex	Male	86 (69%)	9.78 ± 0.49%
	Female	38 (31%)	7.24 ± 0.54%
Body mass index (BMI)	<25	29 (23%)	9.49 ± 0.83%
	25–30	32 (26%)	9.14 ± 0.87%
	>30	63 (51%)	8.70 ± 0.51%
Age	15–30	33 (27%)	10.13 ± 0.83%
	30–65	91 (73%)	8.61 ± 0.44%
Race	Caucasian	49 (40%)	8.30 ± 0.58%
	Hispanic	58 (47%)	9.50 ± 0.64%
	Black	8 (6%)	9.05 ± 1.15%
	Asian	6 (5%)	9.61 ± 1.27%
	Arab	1 (1%)	6.20%
	Unknown	2 (2%)	11.15 ± 0.66%
Hemoglobin A1c (HbA1c)	<5.7%	82 (66%)	8.85 ± 0.49%
	5.7–6.5%	23 (19%)	9.55 ± 0.91%
	>6.5%	18 (15%)	8.87 ± 1.03%
	Unknown	1 (1%)	11.17%
Cause of death	Head trauma	42 (34%)	8.99 ± 0.67%
	CVA/stroke	51 (41%)	8.55 ± 0.65%
	Anoxia/cardiovascular	18 (15%)	11.04 ± 0.95%
	Unknown	13 (10%)	7.97 ± 0.92%

### 3D suspension cell culture

Acinar cells constitute 90% of the pancreas^8^^,^^9^ and are expected to die preferentially compared to ductal cells in the first 24 hours after thawing and plating.^1^ After day 1, it is expected that cells will continue to die, but at a much slower rate.^1^ Spheroids will begin to form between days 3–4 and grow to about 50 μm diameter by day 5–6.^1^ By day 7, it is expected that about 20–30% of cells survive (from the initial cell counts at day 0), and among those surviving cells, all are ductal cells.^1^ Among the ductal cells, 10–25% are colony-forming progenitor cells.^1^

### Frozen embedding of spheroids for immunofluorescence staining

Using this methodology, it is expected that each truncated frozen block contains about 2 mm thick layer containing cells. If cryosectioning is conducted where each cryosection is 10 μm thick and 6 cryosections are placed onto a glass slide, then at least 30 slides can be produced per block. Additionally, using this protocol, most of the antibodies used in Zook et al. 2024^1^ did not require antigen retrieval or permeabilization steps. The use of the Vector TrueVIEW Autofluorescence Quenching kit (SP-8400-15, Vector Laboratories) can reduce background signal, especially in the AF488/FITC channel.

### 3D methylcellulose-based colony assay

After plating, it is expected that by day 4–5 some small cystic colonies will become visible.^2^ Between days 10–18, colonies will continue to grow.^2^ By about day 19–21, the colonies may grow a little more in size, but typically have reached their maximum size of about 200 μm in average diameter^1^^,^^2^; however, there are a range of sizes expected (from 50–2000 μm in diameter).

Depending on the treatment, different colony morphologies may arise. All colonies (>50 μm in diameter) contribute to the overall colony-forming efficiency, but as seen in murine pancreas-derived colonies, different morphologies may contain different cell types.^10^^,^^11^^,^^12^^,^^13^ For individual colony analysis, one can use the methods outlined in Quijano et al. 2023,^2^ Tremblay et al. 2016,^14^ and Kozlowski et al. 2023^10^ to micro-manipulate and examine individual colonies for different cell types that arise under different treatment conditions.

### Processing of colonies and fixation for immunostaining

If plating cells directly from cryostorage in the colony assay medium supplemented with Y-27632, it is expected that the colony-forming efficiency will be around 5%.^1^^,^^2^ If plating suspension cells into the colony assay medium supplemented with Y-27632, it is expected that the colony-forming efficiency will be around 20%.^1^ Additionally, most of the cells within colonies grown using colony assay will be ductal, with some acinar and endocrine cells present.^1^^,^^2^

After washing, fixing, and embedding the colonies, it is expected that each truncated frozen block will contain a ∼2 mm thick layer that contains colonies. Although most colonies collected for immunofluorescence staining are cleared of Matrigel, some colonies may still have residual Matrigel that will emit an autofluorescence background. Thus, including secondary antibody only (negative) controls is highly recommended in order to distinguish the true signal from background or debris. Additionally, including known cell border markers (e.g. CDH1) is helpful to distinguish individual cell morphology.

## Limitations

This protocol has been optimized for primary, islet-depleted human pancreatic exocrine tissues. Tissues from murine sources, pancreatic cells derived from human embryonic stem cells or induced pluripotent stem cells, or primary human pancreatic cells from individuals with pancreatic diseases (e.g. pancreatic cancer or pancreatitis) have not been tested. Additionally, not all human ductal cells survive until day 7 in our suspension culture platform,^1^ suggesting a need to identify additional factors, such as small molecules, that improve their survival. Identification of pro-survival factors will also be important to improve the *in vitro* maintenance of the ductal progenitor-like cells, which comprise on average ∼12% of the total (CD133^+^CD49f^low^) ductal cells from the primary human pancreas.^2^ Lastly, functional beta-like cells can be derived from our methylcellulose-based colony assay^2^ but not yet from the 3D suspension culture system. Therefore, future work will focus on identifying molecules to drive adult human ductal progenitor-like cell activation and differentiation in 3D suspension culture (without methylcellulose and Matrigel).

## Troubleshooting

### Problem 1

For “Preparation of Cryopreserved Adult Human Islet-Depleted Exocrine Cells”, step 3e. The tissue does not settle enough to form a pellet or there are a lot of cells remaining in the supernatant.

### Potential solution

• Although it is expected that not all cells will pellet after 10-20 min on ice (4°C), the supernatant is expected to be mostly clear and a distinct pellet visible.
• An opaque or cloudy supernatant indicates a high number of dead cells, which releases DNA that entangles both dead and live cells. High cell death can be caused by many factors, such as improper storage conditions, long storage time, or rough tissue handling.
• To address whether there are live cells remaining, take a small aliquot from the cloudy supernatant and observe the cells under a light microscope to see whether there are any live cells remaining or if there are just cell fragments. Trypan blue or other live-dead stains or dyes may assist with assessing the potential viability of the tissue preparation.
• If there are still many live cells, add an additional 40–80 μL DNase1 to the 40 mL tissue samples, mix by gentle inversion, and let them sit on ice (4°C) for an additional 10–20 min. Then aspirate the supernatant and proceed. If there are very few or no live cells, but a visible pellet, then proceed with aspirating the dead cell debris in the supernatant and continue the protocol. If there are very few or no live cells and there is no visible pellet, then it is recommended to stop and not proceed with the dissociation.

### Problem 2

For “Preparation of Cryopreserved Adult Human Islet-Depleted Exocrine Cells”, step 4f. The cells re-aggregate and clog the 40 μm filter mesh.

### Potential solution

• If cells clog up the filter, add 10 μL DNase1 stock solution directly to the liquid trapped in the filter cup. Gently pipette the liquid and be careful to avoid tearing the mesh by scraping the pipette tip along it.
• If adding DNase1 does not allow liquid to pass, consider gently lifting the edge of the mesh by the plastic and rocking back and forth to allow any trapped air to release.
• If neither of these options work, consider pipetting out the clump or completely replacing the mesh filter.

### Problem 3

For “3D Suspension Cell Culture: Ductal Cell Spheroid Formation”, step 2l. There are many (>30%) dead cells from the interface of the Histopaque density gradient separation step on Day 1.

### Potential solution

• Dead cells typically pellet at the bottom of the tube or may be suspended in the DMEM/F12/BSA/PS above the cloudy interface (live cell) layer between the DMEM/F12/BSA/PS and Histopaque (Figure 4B).
• To minimize collecting the dead cells, the top 1 mL (or 5 mL if using the 50 mL conical tube) of DMEM/F12/BSA/PS can be aspirated and removed (Figure 4B).
• Additionally, it is recommended to only collect the minimum volume needed to collect the live cell layer. Collecting greater volumes increases the chance of collecting dead cells (Figure 4B).

### Problem 4

For “Frozen Embedding of Suspension Cultured Ductal Spheroids for Immunofluorescence Staining”, step 5h. There are significant remaining ECM proteins coating the cysts/spheroids.

### Potential solution

• Although Cell Recovery solution (Corning, 354253) is effective for removing 5% Matrigel, it may not be as effective for other ECM protein mixtures.
• To remove ECM proteins, the PBS/EDTA solution is an alternative method,^15^ as mentioned in the Processing of Colonies and Fixation for Immunostaining, steps 14g-k.

### Problem 5

For “Frozen Embedding of Suspension Cultured Ductal Spheroids for Immunofluorescence Staining”, step 7c. There is more than 50 μL 30% sucrose remaining with the spheroids/cysts before adding to the OCT.

### Potential solution

• If too much sucrose solution is added to the OCT or if it is not adequately mixed, bubbles or tears can form in the sections during cryosectioning. Therefore, it is important to minimize the amount of sucrose added to the OCT during this step.
• If more than 50 μL of the sucrose solution remains because aspirating the sucrose also aspirates many spheroids/cysts, then one option is to centrifuge and pellet the spheroids/cysts in the excess sucrose solution, then add the pellets together for a final volume of less than 50 μL.
• So long as the final volume added to the ∼2-5 mm OCT is <50 μL, no more OCT should be required.
• If >50 μL sucrose solution is left and centrifugation is not desired, another option would be to add more OCT to the truncated mold prior to adding the spheroids/cysts.

### Problem 6

For “3D Methylcellulose-based Colony Assay: Ductal Progenitor-like Cell Analysis”, step 10j. There are floating cells or cells along the side of the Eppendorf tube or there is significant cell death following Liberase TH and TrypLE digestion.

### Potential solution

• Floating cells and cells sticking along the side of the Eppendorf tube may indicate that cell death is occurring because of the enzymatic digestion.
• If significant cell death occurs, it is recommended to reduce the incubation time of TrypLE.
• It is helpful to observe the cells under a light microscope to determine the status of the digestion. If the cell clusters are dispersed to the stage that individual cells can be distinguished, stop the digestion with FBS at that point.

### Problem 7

For “3D Methylcellulose-based Colony Assay: Ductal Progenitor-like Cell Analysis”. During the 3-week incubation, there is significant pulling of Matrigel and/or too many colonies form.

### Potential solution

• In the colony assay, Matrigel makes a slightly speckled pattern that is distributed evenly across the colony assay medium. If the seeding density was too high and/or if the colonies grew very quickly, this can lead to clear spots or areas surrounding the colony/colonies. Several colonies may be grouped together closely or overlap with one another. In addition, if too many colonies form (>150 colonies per individual well in a 24-well plate), the colonies may run into one another and/or use up the nutrients in the media quickly (resulting in yellow-tinted media by day 21).
• Matrigel pulling and colony overgrowth impact the overall colony counts or colony diameters by day 21. Therefore, it is important to identify whether these effects are occurring by checking on colony growth at least every four to seven days.
• If Matrigel pulling and colony overgrowth occur, repeat the experiment with lower seeding density of colony-forming units. Based on our prior experiences,^1^^,^^2^ it is recommended that the suspension cultured ductal cells are plated at 500–1,000 cells per well, or that freshly-thawed cryopreserved exocrine cells are plated at 2,500–5,000 cells per well. However, different donor samples may require additional seeding density optimization.

### Problem 8

For “Processing of Colonies and Fixation for Immunostaining”, step 13a. Following the three-week incubation, the cell counts of the outside wells (rows A and D) are significantly different compared to those of the inside wells (rows B and C).

### Potential solution

• The evaporation of medium will be uneven between the wells on the outside compared to the inner wells of a 24-well plate. The evaporation will lead to either higher or lower colony growth in rows A and D, compared to rows B and C for unknown reasons. However, in our experience, the difference in colony-forming efficiency between outside and inside wells typically does not exceed 10%, and therefore is not a significant concern.
• If the difference between outside and inside wells is >10%, first check the incubator for its water level needed for sufficient humidity. Additionally, the culture plate can be placed inside a covered tray that contains open petri dishes containing sterile water to enhance humidity within the tray.
• If the counts increase or decrease steadily from row A to row D, this may indicate that the mixing of the cells with the colony assay medium prior to plating was not thorough enough. It is recommended that the mixing of the cells in the 5 ml polystyrene snap-cap tube be done with vigorous shaking for ∼30 s, but another method to promote adequate cell mixing before plating is to use the syringe to mix the media prior to dispensing into the wells.

### Problem 9

For “Processing of Colonies and Fixation for Immunostaining”, step 14h. The fixed colonies still have significant amounts of Matrigel-methylcellulose mixture sticking to them.

### Potential solution

• Some of the Matrigel-methylcellulose mixture may remain stuck to the colonies, owing to the tight cell-ECM protein interactions and binding that occurs. Remaining ECM proteins and/or methylcellulose can lead to autofluorescence signal during immunofluorescence staining and imaging. However, in our experience, the PBS/EDTA wash detailed in the above protocol removes most of the mixture without compromising the colony morphology.
• If there are still significant amounts of mixture remaining, this may indicate inadequate agitation during the PBS/EDTA wash step. To address this, one can incorporate a step to gently invert the tubes 3-5 times every 10 min or adjust the rocker/shaker speed to increase agitation of the samples. Too much agitation may lead to breakage of colonies into smaller fragments, however.

## Resource availability

### Lead contact

Further information and requests for resources and reagents should be directed to and will be fulfilled by the lead contact, H. Teresa Ku (hku@coh.org).

### Technical contact

Technical questions on executing this protocol should be directed to and will be answered by the technical contact, Heather N. Zook (hzook@coh.org).

### Materials availability

This study did not generate new unique reagents.

### Data and code availability

The published article^1^ includes all datasets generated or analyzed during the original study.

## Acknowledgments

This work is supported, in part, by grants from the National Institutes of Health to H.T.K. (R01DK099734 and R56DK099734) and the City of Hope Research Facilities (P30CA33572). H.N.Z. is a recipient of a Postdoctoral Fellowship from the California Institute for Regenerative Medicine (CIRM). Support from the Wanek Family Project for Type 1 Diabetes and from an anonymous donor, S.S., to H.T.K. is also gratefully acknowledged. We would like to thank the services provided by the following research cores at the City of Hope in the preparation of this methods article: Analytical Flow Cytometry, Light Microscopy Digital Imaging, and Biostatistics. The graphical abstract and many figures were prepared using BioRender.com.

## Author contributions

Conceptualization, H.N.Z. and H.T.K.; methodology, H.N.Z., J.C.Q., J.A.O., and C.D.; investigation, H.N.Z., J.C.Q., J.A.O., and C.D.; validation, H.N.Z., J.C.Q., J.A.O., C.D., and N.E.; writing – original draft, H.N.Z. and H.T.K.; writing – reviewing and editing, all authors; funding acquisition, H.N.Z. and H.T.K.

## Declaration of interests

H.T.K. is an inventor of the patent no. 9,783,784, titled “Methods for establishing and improving the survival of a population of pancreatic progenitor or stem cells.”
